# Modelling the Spread of Farming in the Bantu-Speaking Regions of Africa: An Archaeology-Based Phylogeography

**DOI:** 10.1371/journal.pone.0087854

**Published:** 2014-01-31

**Authors:** Thembi Russell, Fabio Silva, James Steele

**Affiliations:** 1 School of Geography, Archaeology and Environmental Studies, University of the Witwatersrand, Johannesburg, South Africa; 2 Institute of Archaeology, University College London, London, United Kingdom; 3 School of Archaeology, History and Anthropology, University of Wales Trinity Saint David, Lampeter, United Kingdom; University of Oxford, United Kingdom

## Abstract

We use archaeological data and spatial methods to reconstruct the dispersal of farming into areas of sub-Saharan Africa now occupied by Bantu language speakers, and introduce a new large-scale radiocarbon database and a new suite of spatial modelling techniques. We also introduce a method of estimating phylogeographic relationships from archaeologically-modelled dispersal maps, with results produced in a format that enables comparison with linguistic and genetic phylogenies. Several hypotheses are explored. The ‘deep split’ hypothesis suggests that an early-branching eastern Bantu stream spread around the northern boundary of the equatorial rainforest, but recent linguistic and genetic work tends not to support this. An alternative riverine/littoral hypothesis suggests that rivers and coastlines facilitated the migration of the first farmers/horticulturalists, with some extending this to include rivers through the rainforest as conduits to East Africa. More recently, research has shown that a grassland corridor opened through the rainforest at around 3000–2500 BP, and the possible effect of this on migrating populations is also explored. Our results indicate that rivers and coasts were important dispersal corridors, but do not resolve the debate about a ‘Deep Split’. Future work should focus on improving the size, quality and geographical coverage of the archaeological ^14^C database; on augmenting the information base to establish descent relationships between archaeological sites and regions based on shared material cultural traits; and on refining the associated physical geographical reconstructions of changing land cover.

## Introduction

In just a few thousand years farming spread from a cradle in West Africa to cover an area of more than 23 million square kilometres of sub-Saharan Africa, occupied today by more than 200 million Bantu language speakers speaking approximately 440 to 680 different Bantu languages [1]. It has been hypothesised that farming and Bantu languages dispersed simultaneously through demic expansion [2], [3], [4], [5], [6], [7], [8]. This is debated in archaeology, where there are proponents of demic expansion, cultural adoption-diffusion, and demic diffusion explanations [9], [10], [11], [12], [13], [14], [15]. A literature review reveals however that demic expansion is still the overwhelmingly favoured explanation (see Table S1). Working within the demic expansion/demic diffusion framework, this paper describes new models of the spread of these farming populations from an origin in West Africa under different possible sets of environmental constraints, conditioned by archaeological evidence from a newly-compiled geo-referenced radiocarbon database.

Linguistic and archaeological evidence places the cradle of Bantu-language speakers in the Nigeria-Cameroon border area [7], [16] and it is from here, that the expansion of pottery-making Neolithic Bantu-speaking horticulturalists/farmers started, with archaeologists finding apparent evidence for an early ‘deep split’ into two branches: the Eastern Bantu and the Western Bantu [6], [7], [16], [17]. The earliest pottery found in a Bantu-speaker area is that from the site of Shum Laka in north-western Cameroon, dating to perhaps as early as 7000 BP [18], [19], [20], [21], [22]. Western Bantu expansion happened southwards from here, with the area to the west of the Sangha River, in the Democratic Republic of Congo, being settled first [16]. The pottery traces of the southward expansion are found at Obobogo in Cameroon; at the Denis 1 and 3 Settlements in Gabon (5000–3000 BP); at Nzogobeyok in Gabon (4800–4400 BP) and at the sites of Okala, Ndjolé, Kango, Lalala, Mindoubé, Inkengué, Mbilapé and Lopé in Gabon (2600–2400 BP) [18]. The pottery is found in similar contexts to the Shum Laka pottery; in association with the remains of village settlements, polished stone tools such as axes and hoes, upper and lower grinding stones, grooved stones, charcoal, quartz debitage, evidence of palm tree cultivation and the grains of the *Canarium schweinfurthii* [1], [16], [18], [19]. Yams, which may also have been cultivated, leave no archaeological trace [16]. These early farmers were neither smelting nor using metal [16], [18], [19], [20], [23], [24]. The expansion of the Western branch southwards and south-eastwards through Central Africa continued as far as the present Zambia-Malawian border, the Zambia-Zimbabwean border and the Namibian-Angolan border [6], [7]. The oldest pottery found at the site of Benfica in Angola, dating to *circa* 200 AD is similar to that found on the more northerly Neolithic Bantu-speaker sites [1].

The environment through which the farmers moved provided both accelerators and obstacles to their movement. The great swamps and marshes of the Congo rainforest, the arid Batéké plateau on the border between Gabon and the Republic of Congo and the Du Chaillu massif in Gabon, were unsuitable for habitation and had to be circumnavigated [16]. Conversely, the shoreline and rivers provided corridors for rapid movement [12], [25]. Vansina [16] proposes that an initial rapid expansion southwards by sea carried a group from Cameroon to Gabon. Similarly, Blench [26], using linguistic data, proposes that there was a maritime expansion of Bantu speakers along the West coast. Clist [18] has suggested that in Gabon, it is likely that the Ogooué River “was a major diffusion and migration axis”. Phillipson [7] also emphasises rivers and coastal routes in the initial migration of farmers to the south-western margin of the rain forests of west-central Africa. In terms of settlement choices, Vansina [16] suggests that forest-savanna ecotones were especially favourable for the type of root and tree crop cultivation practiced by the western Bantu-speakers.

In Phillipson’s version of the ‘deep split’ model, an Eastern population stream, from an origin in Cameroon, spread along the northern margin of the rainforest to reach the inter-lacustrine region of East Africa. He suggests that it was during this spread that contact with more northerly non-Bantu groups led to their adoption of domestic livestock and the acquisition of metal working skills and knowledge, although more recent work has suggested alternative hypotheses for the appearance of metal working and herding [12], [18], [27], [28]. In this account, during the first millennium AD Bantu-speaking farmers spread through eastern and southern Africa from the inter-lacustrine region. Archaeologically they are distinct from the western Bantu speakers, and are recognised by their pottery, the use of iron, domesticated livestock herding and cultivation of cereal crops such as sorghum and millet (this ‘package’ was termed the Chifumbaze complex by Phillipson [7] and is also known as the Early Iron Age Industrial complex). It first appears with Urewe pottery in the Great Lakes region from about 2500 BP onwards [1], [7]. Occurring in areas where eastern Bantu languages are spoken today, this is seen as the archaeological trace of their arrival [6], [7]. The eastern stream links, through pottery typology, the great Lakes region in East Africa to KwaZulu-Natal in South Africa [6], [7].

In a refinement of Phillipson’s [7] two stream model, Huffman [6] proposes a three stream model, with the addition of a Central stream. Huffman’s Central stream contains sites that Phillipson had classified as Western stream & which he correlated to the spread of the Urewe makers southwards and south-westwards from the inter-lacustrine area around the bottom of the rainforest. Here they meet the southward expanding Western stream Bantu speaking farmers. This coalescence then gave rise to the Western stream of the Early Iron Age Industrial Complex that expanded into Angola, Namibia and south-eastwards towards Zambia and Zimbabwe [6]. The idea of contact between the inter-lacustrine Urewe Bantu-speakers to the east and the Bantu-speakers to the West is discussed by Digombe *et al* [29]. They think that the only close parallel to the type of iron furnaces found in Gabon are with those found to the east in the inter-lacustrine region. However, no trace of iron or of pottery similar to the Urewe pottery of the inter-lacustrine region, and therefore suggestive of such contact, is found anywhere along the connecting route between these two areas, and so there is caution about claiming such a link based on the available evidence [1], [18], [30].

These archaeological models of two or three streams are widely cited, and seen by many archaeologists as supporting a parallel ‘deep split’ in the radiation of the Western and Eastern Bantu languages; but they need to be evaluated in relation to physical anthropological [31], genetic and linguistic data, as well as continuing archaeological discoveries. Some recent phylogenetic work in linguistics and genetics does not find support for such a ‘deep split’ [32], [33], [34]. Instead these studies find support for some version of a ‘pathway through the rainforest’ scenario, with the Eastern Bantu language clade radiating much later in time. It is useful therefore to re-examine the archaeological evidence in light of these new genetic and linguistic results, to see whether an independent phylogeography based on archaeologically-observed arrival times also supports a ‘late split’ [33] for Eastern from Western Bantu with a primary dispersal route southward through the rainforest preceding that split [34].

In archaeology, a standard way of reconstructing dispersal routes and dispersal chronology for radiations such as those of the early farming cultures is to compile archaeological radiocarbon dates for their first observed occurrences throughout the geographical region of interest, and to look for spatial gradients in arrival times. Statistically such trends can be evaluated with regression techniques, typically by bivariate analyses of the relationship between observed arrival time and distance from some origin point [35]. Such trends can then be used to estimate parameters for reaction-diffusion models in the Fisher-Skellam tradition, as a constraint on demographic hypotheses of the spread dynamic [36], [37], [38]. This approach has been used to study the spread of farming into Europe across the prehistoric Neolithic transition [39], [40]. In recent work, attention has focused on developing methods to evaluate the effects of terrain, drainage, and biome type on dispersal rates across different landscapes [41], [42], as well as to evaluate the congruence of archaeological models with estimates of dispersal paths and timescales derived independently from other kinds of data (e.g. genetics, [43] and classically, [44]).

In this paper we use such methods to reconstruct the dispersal of farming into areas of sub-Saharan Africa now occupied by Bantu language speakers. Literature review identifies several suggestions for how environmental variables may have influenced spread rates, and these are explored in our modelling. The ‘deep split’ hypothesis suggests that the eastern Bantu stream spread around the northern boundary of the rainforest and that forest/savanna boundaries were particularly attractive to the first farmers. An alternative riverine/littoral hypothesis suggests, in contrast, that rivers and coastlines facilitated the migration of the first farmers, with some extending this to include rivers through the rain forest as conduits to East Africa. More recently, research has shown that a grassland corridor opened through the rainforest at around 3000–2500 BP [45], [46], [47], [48], so the possible effect of this on migrating farmers is also explored. We explore these alternative scenarios below, and also introduce a less constrained way of exploring the wider parameter space that enables some of their elements to be combined.

## Materials and Methods

### 1 Materials

#### The database and its compilation

The database contains geographically referenced radiometric determinations that by their association with archaeological material (most commonly pottery) are interpreted by the excavator/archaeologist as marking the first arrival of Bantu language speaking farmers to an area. Data were collected from those countries in sub-Saharan central, eastern and southern Africa where Bantu languages are spoken today. The database was compiled from a combination of site reports, academic publications, radiocarbon laboratory lists and existing databases both in print and online [49]. 804 records have complete entries (i.e. both coordinates and radiometric dates): 794 radiocarbon determinations and 8 thermoluminescence dates from 331 archaeological sites. Calendar ages for the earliest farming-related occupation of each such site were obtained by radiocarbon calibration in OxCal 4.1 using the IntCal09 calibration curve [50], [51]. Where multiple dates had been obtained at a site and they were close enough in age to be potentially derived from a single occupation event [52] they were averaged prior to calibration; otherwise, we used the oldest date in any such site-specific series. We then took the mean calibrated age as a point value in time for each site (calendar years BP), as an input into our modelling. To improve the accuracy of the analysis by reducing the “noise” provided by sites that do not correspond to first observed arrivals in their neighbourhood; the dataset was then filtered using an iterative two-dimensional binning technique to select the oldest site in a given neighbourhood. A neighbourhood radius was set at 100 km, which we considered to be a minimum spatial separation required for resolving by radiocarbon dating any evidence of a diffusing front moving at c.1 km/yr (the order of speed typically estimated for prehistoric human dispersals in the existing literature, [44], [53]. Only the oldest dated sites in each neighbourhood were retained for further analysis. This reduced our initial sample of 331 dated sites to 108 retained for further analysis (see table S2, figure S1 and text S1).

#### Base-maps used in the analysis

To define land/sea boundaries we used a present-day world coastlines map, projected using the Lambert Azimuthal Equal Area projection (centred at 10°S, 25°E). This is an appropriate projection for the domain of interest, which is predominantly tropical, with a north-south orientation. To define land cover classes we used the biomes in the 2004 version of the Terrestrial Ecoregions of the World shapefile, compiled by Olson *et al* [54], with limited further aggregation of biome types (see figure S3 and details in SI). The savanna corridor was created based on the reconstructed maps given by Maley [45] (see figure S4). In addition, the Congo and Zambezi rivers, and their major tributaries, were taken from the ESRI World Rivers shapefile. These two drainage basins were considered separate features to enable the Congo to be a corridor through the rainforest if needed. Other major African rivers were not considered relevant for this initial study.

### 2 Methods

#### Regression analysis

For our modelling, which requires an approximate origin point, we have chosen a point in northwest Cameroon at 5°51′N, 10° 4′E, close to the site of Shum-Laka (the oldest site in the database). The statistical methodology used to estimate trends in earliest observed arrival dates as a function of distance from the assumed origin, involved fitting regression models (reduced major axis [35], [55]) to sets of paired values of site dates (mean calibrated radiocarbon ages, calibrated in OxCal using INTCAL09) and distances to sites from the assumed origin. Using regression slopes to estimate average front speeds is established practice in the literature [35]. This enabled us to estimate the mean speed of dispersal (using the regression slope coefficient), and the proportion of the variation in arrival times that was accounted for by that trend (using the correlation coefficient). We estimated (using the correlation coefficient) best-fitting speeds of dispersal in different directions as a function of habitat, with coasts, rivers, and major ecoregions all being given individual values for their possible effects on rates of spread. We modelled these effects using Matlab code written especially for this purpose, but which approximates in key respects the algorithms found in GIS modules that perform a raster cost surface calculation. In future work it would be desirable to estimate the effect on such models of geographical variation in the density of archaeological coverage (i.e., do less well-studied areas tend also to yield younger ages for first observed settlement, contributing to a significant recovery bias?), see [56].

#### Two explicit models tested

We tested the fit of two scenarios from the literature. Phillipson’s [7] suggestion that the eastern Bantu stream spread around the northern boundary of the rainforest and Vansina’s [16] hypothesis that the forest/savanna boundaries were particularly attractive to the first farmers can be combined into a ‘Deep Split’ model according to which coastlines and the forest/savanna boundary should be easy to disperse along, but major rivers and rainforest should be hard to cross (and savanna moderately hard). Subject to these relative ease-of-dispersal constraints, we then systematically explored the parameter space for possible values for rates of dispersal through each of these categories of geographic corridor and major biome. To test an alternative ‘Rivers and Coasts’ hypothesis that rivers and coastlines facilitated the migration of the first farmers (with some authors extending this to include rivers through the rainforest acting as conduits to East Africa), we specified a ‘Rivers and Coasts’ model in which major rivers, coastlines, and the savanna biome should be easy to disperse across, but rainforest should be hard (and other forest moderately hard). Subject to these relative ease-of-dispersal constraints, we again then systematically explored the parameter space.

#### Obtaining the dispersal parameter set that best fits the dataset

In order to let the archaeological dataset speak for itself and not impose any prior constraints on the models, we also attempted to obtain the parameter set (i.e. the set of cost factors for each ecoregion and water corridor) that provides the best fit to the radiocarbon dataset independent of prior hypotheses in the literature. The problem is one of optimization, i.e. of finding the set of parameters that maximizes a fitness function: in this case the correlation coefficient. Fully exploring the parameter space is a computationally slow process so, in order to quickly and effectively to find the best-fit model we decided to implement in Matlab a Genetic Algorithm (GA henceforth; see text S1). GAs start with a random population of models (i.e. a set of models with random values for the parameters) whose fitness is evaluated by some function (in our case the distance vs arrival time correlation coefficient). The best-fit models are then copied to the next generation unscathed (cloned), whereas less fit models are discarded. To keep the population size constant, the best-fit models are also allowed to reproduce. This involves the genetic principle of crossover, in which both parent models give only a part of their parameter set to the child model. Mutation can then occur on any model of the new generation, except for the very best one. Crossover and mutation are controlled by fixed rates and are essential to ensure that the GA does not get stuck on a local maximum of the fitness function but instead samples enough of the parameter space to lock onto a global maximum. This process is iterated several times until a certain condition is met. After several generations the population begins to converge on the parameter set that maximizes the correlation coefficient.

#### Using least-cost paths to create dispersal trees

The regression modelling yields, for each solution, a cumulative cost surface which can be rescaled to show mean expected arrival times in years BP. Least-cost paths can then be traced back from each point in the archaeological dataset to the origin point; points where least-cost paths meet can be treated as nodes on a tree, and the entire least-cost path network represented as a phylogenetic “dispersal tree” whose topology can be extracted from the map and represented in more conventional diagrammatic form (using native Matlab code; see text S1). Clades can then be shaded for pottery style variants or other cultural attributes, to assess congruence between the model solution and the splits inferred from these other independent sources. Similarly, having obtained a well-fitting archaeological map of predicted arrival times conditioned by radiometric dates, congruence can then be examined with language phylogenies by extracting an archaeologically-modelled dispersal tree in which the branches end at the centroids of modern language areas, rather than archaeological sites.

## Results

### Isochron Mapping

**Figure 1:**
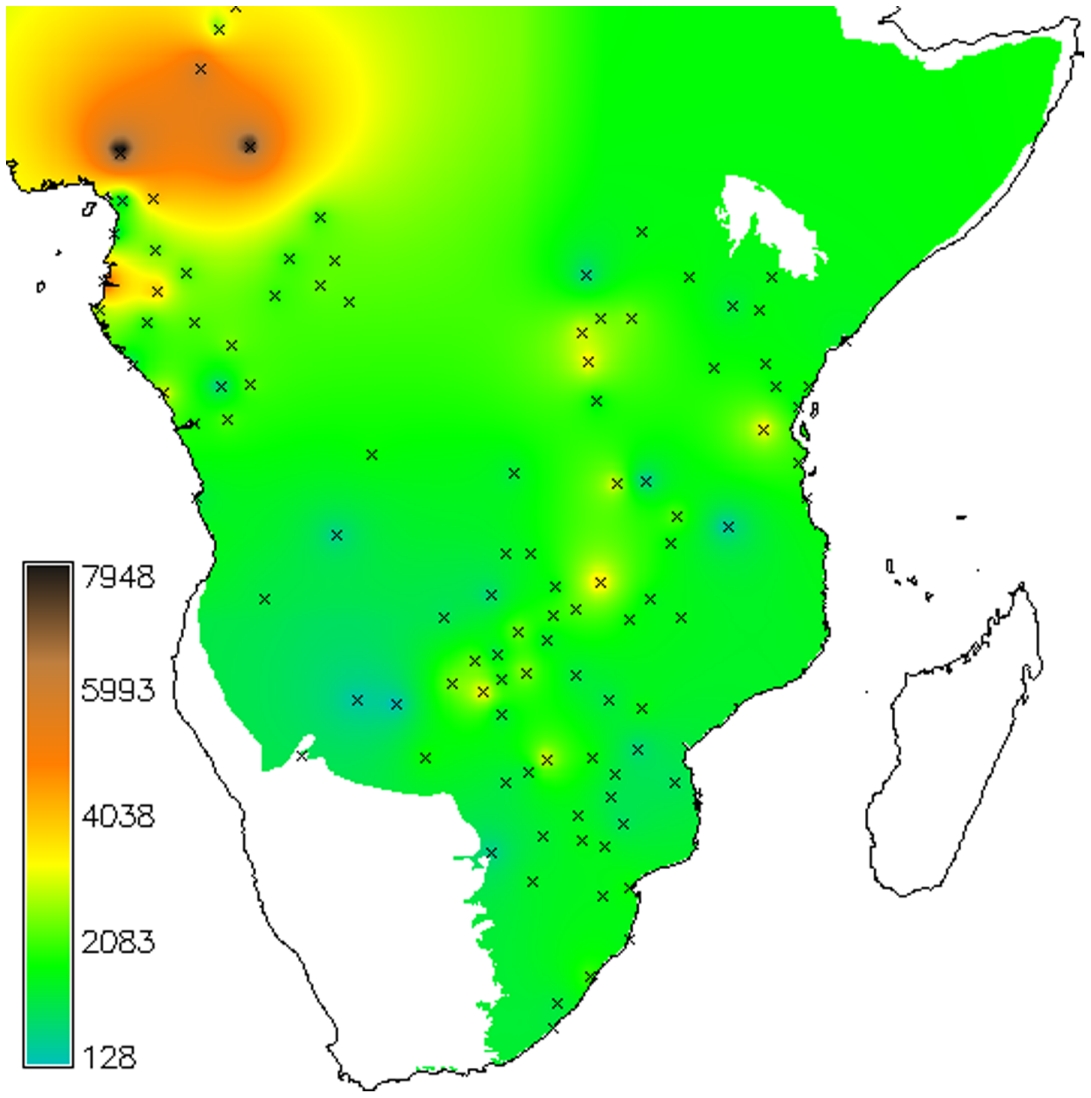
Archaeological sites retained after binning, with interpolated age contours (cal BP).

As an initial visualization, a contoured isochron map of observed arrival times was generated in GRASS using bilinear interpolation with Tykhonov regularization (Figure 1 and additional Figure in SI; the routine is *r.resamp.bspline,* with lambda = 0.01).

This shows that from the evidence currently available, farming spread slowly within the Cameroon region, between 7,000 and 4,000 years BP, with some sites showing up in Eastern Africa by 3000 BP. Between 3,000 and 2,000 BP farming is found more widely, with farming reaching southern Africa, while by 1500 BP there is a clear signal in the northeast of South Africa/southern Mozambique (see figure S2). There is reason to believe that the map is influenced by research effort: in Zambia and Zimbabwe, where there is a greater density of dated sites (Figure 1 and table S3), there are also earlier sites than in neighbouring countries; while the gap in coverage along coastal northern Mozambique may explain the seemingly late appearance of farming in that long part of the eastern coastal region.

We then used our modelling framework to obtain the best-fitting parameter sets for the two explicit scenarios, and also for the unconstrained search using genetic algorithms for two different ecoregion basemaps (one with and one without the reconstructed savanna corridor through the equatorial rainforest, [45]). The genetic algorithms yielded better-fitting solutions than either of the pre-specified models, even after controlling for their extra degrees of freedom (Table 1).

**Table 1:** Fitted dispersal speeds (km/yr), and statistics for each ecoregion and corridor, for the two pre-existing models and for the best-fit GA models with and without savanna corridor.

Speeds (km/yr) on:	‘Deep Split’ Model	‘Rivers and Coasts’ Model	GA best-fit w/o corridor	GA best-fit w/corridor
**Congo River**	0.09	0.98	0.31	1.39
**Zambezi River**	0.88	0.20	5.28	4.32
**Coasts**	1.76	0.98	0.36	0.03
**Rainforest**	0.09	0.10	0.09	0.05
**Savannah**	0.88	0.98	0.66	0.39
**Forests**	0.88	0.20	5.28	3.69
**Rainforest Boundary**	0.88	N/A	0.14	0.03
**Montane**	0.88	0.20	0.11	0.11
**Pearson’s correlation coeff. r**	−0.521	−0.5075	−0.639	−0.669
**Aikaike Information Criterion**	440.74	439.50	434.14	430.13

**Figure 2:**
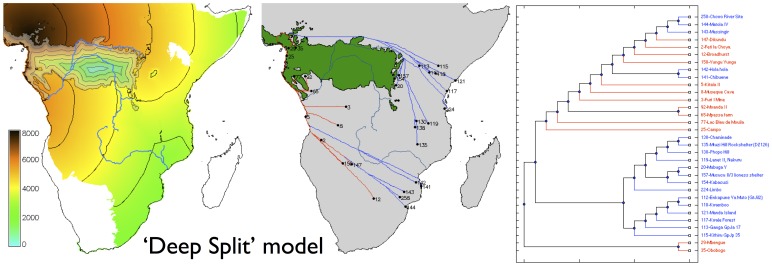
Modelled arrival time surface (left), least-cost path network (centre) and corresponding dispersal tree (right) for the ‘Deep Split’ model. Contours at 1,000

**Figure 3:**
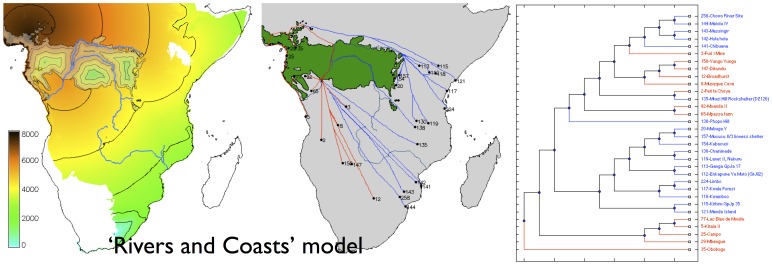
Modelled arrival time surface (left), least-cost path network (centre) and corresponding dispersal tree (right) for ‘Rivers and Coasts’ model. Contours at 1,000

For the pre-specified ‘Deep Split’ model, the best-fitting parameter set (Figure 2) is one in which there was rapid dispersal (c. 2 km/yr) along the coastline of the regions suitable for farming, with much slower dispersal through the rainforest and along the Congo (less than 0.1 km/yr). The Western and Eastern Streams converge with a boundary in southern Mozambique. For the ‘Rivers and Coasts’ model, the best-fitting parameter set (Figure 3) is one with rapid dispersal along the Congo, coasts, and across the savanna biome (c. 1 km/yr, compared with 0.1 km/yr for the rainforest and c. 0.2 km/yr for the other major rivers, notably the Zambezi). The model we obtained predicts a major contribution by the Western Stream with rapid dispersal along the rivers of the Congo basin, and with a boundary between the Western and Eastern streams near the border between Tanzania and Mozambique.

**Figure 4:**
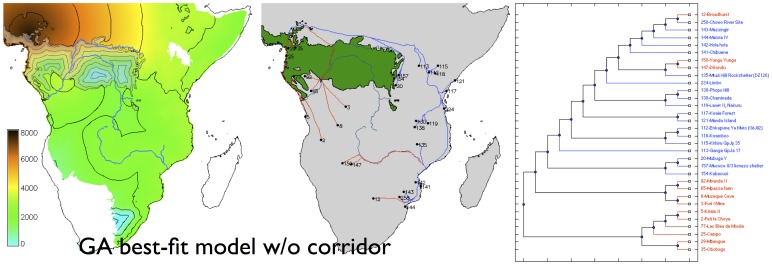
Modelled arrival time surface (left), least-cost path network (centre) and corresponding dispersal tree (right) for the best-fit model without a savanna corridor. Contours at 1,000

**Figure 5:**
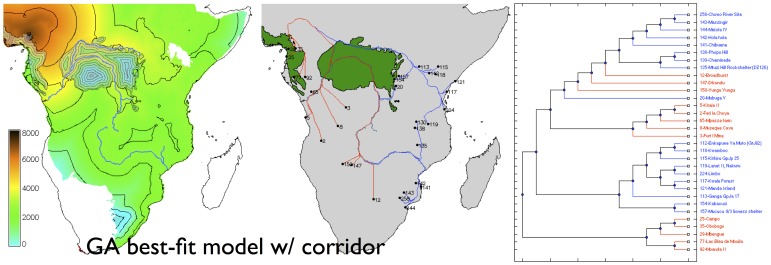
Modelled arrival time surface (left), least-cost path network (centre) and corresponding dispersal tree (right) for the best-fit model with a savanna corridor. Contours at 1,000

With a fuller exploration of the parameter space unconstrained by pre-existing models in the literature, the genetic algorithms found two significantly better-fitting, but very contrastive, scenarios (Table 1). With no savanna corridor through the rainforest, we recover the Deep Split scenario traditionally favoured by archaeologists, with the Eastern Stream dominant, dispersal proceeding along the Zambezi inland from an east coastal starting point, and a boundary between the Eastern and Western streams in southern DR Congo and eastern Angola (Figure 4). However, with a savanna corridor implemented, we find that it is a Western/Central Stream that is dominant, with dispersal downstream along the Zambezi towards the east coast, and with the boundary with the Eastern stream in Tanzania (Figure 5). The latter model is the best-fitting of the two, although in all cases the correlation coefficients indicate that more than half the variance in archeologically observed arrival times remains unexplained (Table 1).

As independent evidence of the goodness of fit of each of these models to the archaeological data, we can also ask how well the dispersal trees segregate the sites in the database into the ‘Streams’ to which they were assigned based on pottery typology (after Phillipson [7], Huffman [6]; although there have been challenges to interpretations of the ceramics upon which the Eastern Stream is traditionally modelled [12], [57]). Comparing the ‘Deep Split’ and ‘Rivers and Coasts’ models (Figures 2 and 3) we see that while the correlation coefficients for the best-fitting parameter sets are almost identical, the dispersal tree for the ‘Deep Split’ segregates sites into clades which visually correspond better with the pottery-based Streams; this suggests that this scenario is likely to be the better reconstruction. Similarly, when we compare the two best-fitting models obtained using genetic algorithms (Figures 4 and 5), each again having a very similar value for the correlation coefficient, the scenario with no savanna corridor (another ‘Deep Split’ scenario) provides a visually better fit to the Streams reconstructed from pottery typology. However, the palaeoecological evidence for the corridor is increasingly unambiguous, and this gives independent support to the best-fitting model obtained with a savanna corridor implemented.

**Figure 6:**
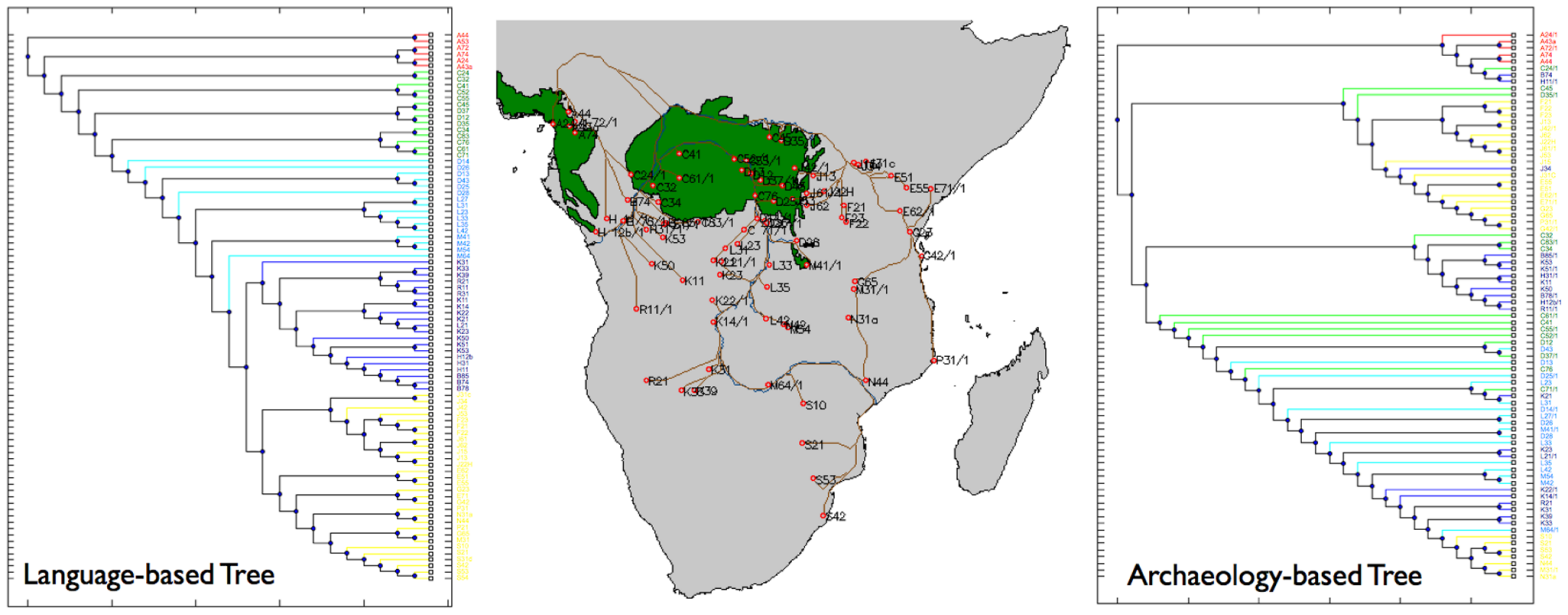
Rexová et al. [32] language-based tree (left) compared to the shortest path tree obtained from the model that best fits the archaeological data (GA with savanna corridor), with language area centroids as the terminal points of the least cost paths (centre and right).

To test for congruence between the radiocarbon-based dispersal models and other independently derived models, one can use the dispersal tree methodology to make comparisons. One can use the centroids of language distributions to reconstruct the archaeological shortest path tree of those populations for a given dispersal model, and compare it to trees independently derived from lexical data. In the following we have extracted the geographical coordinates for the Bantu language centroids from [58], created the shortest path trees predicted by the dispersal model that best fits the radiocarbon dataset (the GA solution with savanna corridor implemented), and compared it with the maximum parsimony tree of 87 languages produced by Rexová *et al.* [32] (in future work we will examine other language trees similarly, e.g. [34]). Figure 6 allows for a visual comparison between these two independently derived trees. The colouring of the branches follows Rexová *et al*’s groupings into: initial radiation (red), branching in the rainforest (green), main radiation (light blue), westward spread (dark blue) and migration to eastern and southern Africa (yellow). The colouring of the branches was maintained for both trees to facilitate comparison.

The archaeology-based phylogeographic tree of Bantu languages does not display as tight a clustering of languages as the lexicon-based tree. However, some of its general trends are also present. Guthrie Zone A languages form an initial radiation group (red). In the archaeological tree these are joined by three other languages, whose centroids are located at the southern end of the savanna corridor. After this initial radiation event the archaeological tree features a split into two main branches, corresponding to a split between a shortest path that follows the Ubangi/Congo southward and then eastward, and one that follows the upper Ubangi river and northern forest/savanna edge eastward. This contrasts with Rexová *et al.* [32], whose tree features a rainforest branching (green) and main radiation (light blue) before the split between the traditional western and eastern branches. Because the split occurs earlier in the archaeology-based tree, most of the green and light blue languages fall inside one of these branches, particularly the Congo one, and are not as perfectly structured as in the language tree. The Congo branch further bifurcates into a branch leading towards the western coast, and a central branch following the Congo-Zambezi drainage basin towards the southeast. Due to the Zambezi corridor effect, which effectively links the Congo basin with the southeast, Guthrie zone M, N and S languages, traditionally attributed to the eastern branch and thus coloured yellow, split off this branch and are thus separated from the Ubangi group. The core of the latter consists of Guthrie zone J, F, E, G and P languages.

## Discussion

Archaeologists have long emphasised the possibility of deep split in the dispersal history of first farmers in the Bantu-speaking regions, a view that has been partly conditioned by early dates in the interlacustrine region of east Africa. Linguists reconstructing dispersal history from language phylogeny have however increasingly favoured a ‘pathway through the rainforest’ model, with a much later branching of the Eastern Bantu language groups. Geneticists have similarly found evidence against a deep split [33], [59], although others also recognize that the genetic evidence points to a much more complex picture than either a single or an early-bifurcating wave of advance [60], [61].

A full resolution of the question of dispersal routes and rates will not be attained until we have fuller and more balanced geographical data on arrival times. This paper is the first attempt to compile a geo-referenced database of archaeological remains associated with the spread of the first Bantu-speaking farmers in sub-equatorial Africa. The challenge to such archaeological database building remains the reliability of the association between the dated material and the event under question; in the present analysis, this was guided by the individual excavators’ expert interpretations. As more data become available it would be useful to separate records according to what is being dated (animal stock, floor remains, plant remains etc.); this is not yet possible due to the paucity of data, and the database is predominantly pottery-based. An early Iron Age metals database [25] provides the potential for a parallel analysis. There is an obvious bias in the dataset to countries where a great deal of fieldwork and dating have been undertaken, notably Zambia, Zimbabwe and South Africa. There are major gaps in the data from regions such as Angola, the Democratic Republic of Congo and Mozambique that are likely to affect the model outcomes. Site numbers per land area per country in the full, unfiltered dataset highlight the problem (table S2 and figure S1).

Our models make it clear that geography affected dispersal rates: we found effects of corridors, barriers, and of different habitat types. Our GA-optimized results further emphasize the importance of accurate geographical reconstruction, with a key role found for a now-vanished late Holocene savanna corridor through the equatorial rainforest. Future work could usefully explore the sensitivity of such results not just to improved archaeological chronologies but also to different scenarios of dynamically changing vegetation, gradual or abrupt.

To illustrate the dependence of our results on archaeological data and on the modeling assumptions, consider the case of the Congo river and its tributaries. The Ubangi is the largest right bank tributary and it leads fairly directly eastwards (via its own Uele tributary) towards the northern end of the African Great Lakes region. Any geographical model that allows for rapid dispersal along the Congo system will inevitably reconstruct a split between the dispersal pathways following part or all of the Ubangi and those following the main Congo branch leading to the southeast and to the Zambezi. Archaeological data can help determine whether or not such a dual-corridor scenario is justified, but only if the dates and cultural affiliations are well-resolved, and here as well, much more work is needed. At present the earliest dated ceramics from the Ubangi corridor are of the Batalimo-Maluba type, dated to about 1900 cal BP at Maluba [62], which is later than predicted by the best-fitting model; but the region is archaeologically not yet well-explored. It may be therefore that in future, targeted fieldwork can be done to test hypotheses about earliest settlement along dispersal corridors and the results used to constrain further rounds of modeling.

The Zambezi River also emerges as an unexpected corridor in our GA-optimized models. There is archaeological evidence that the Zambezi and environs would have been a favourable corridor for farmers. Posnansky [3] postulates that a major expansion of farmers might have occurred from the Zambezi-Congo watershed. A preference for riverine settlements amongst early farming communities is described by Pwiti [63], in his study of early farming settlements in the mid-Zambezi valley, Zimbabwe. He suggests that rivers were attractive because of the good agricultural soils and access to water. Other riverine resources, such as fish, clay and game might have also made these areas attractive. Similarly, in his study of early farmer settlements in the Tugela River Valley, South Africa, Maggs [64] notes a preference by early farmers to settle along river valleys. Early farming communities in Zambia too, are also located close to rivers [65]. In his general discussion of the spread of farmers, Vansina [12] makes the observation that Bantu languages spread by major river routes, and as noted in the introduction, others too have hypothesized that rivers facilitated migration and diffusion [7], [16], [18].

In conclusion, we have compiled a new database of archaeologically-observed arrival times for the first farmers in the Bantu-speaking regions, and have developed a suite of methods to use this database to estimate dispersal routes. We have also introduced a method of modelling phylogenetic trees from archaeological data that can be used to assess congruence with phylogenies reconstructed independently from genetics and linguistics. Our results are consistent with more than one dispersal scenario, and highlight the opportunity for targeted archaeological work in sparsely-sampled locations (table S2 and figure S1) to help resolve remaining ambiguities.

## Supporting Information

Figure S1 **Distribution of all sites in database, with those that remain for analysis after 2D binning shown in red.**
(TIFF)

Figure S2 **Isochron surface of the filtered subset.**
(TIFF)

Figure S3 **(left) All Olson biomes for the domain of interest; (right) biomes used for modelling, after aggregation.**
(TIFF)

Figure S4 **Map of the rainforest with savanna corridor, following Maley (2002).** Also represented are the Congo and Zambezi rivers, and major tributaries, used in the modelling algorithms.
(TIFF)

Table S1 Mechanisms of dispersal of farming and Bantu-languages in sub-Saharan Africa and how they are recognised, by Author.
(DOCX)

Table S2 Database used in the analysis, after the site selection process.
(DOCX)

Table S3 Number of sites and dates on the full database, per country and area.
(DOCX)

Text S1 **Supporting information on methods used.**
(DOCX)
