# Supplementary figures and images for: Modelling the Spread of Farming in the Bantu-Speaking Regions of Africa: An Archaeology-Based Phylogeography
Source: PLoS One. 2014 Jan 31;9(1):e87854. doi: 10.1371/journal.pone.0087854 (PMC3909244; doi:10.1371/journal.pone.0087854)

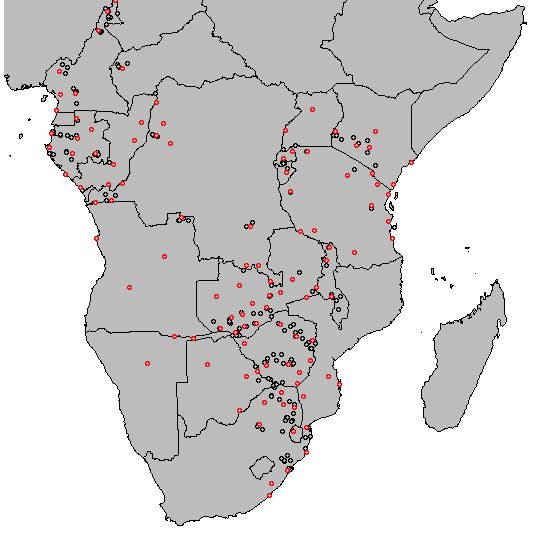

Supplement: Figure S1 — Distribution of all sites in database, with those that remain for analysis after 2D binning shown in red. (TIFF) [file pone.0087854.s001.tiff]

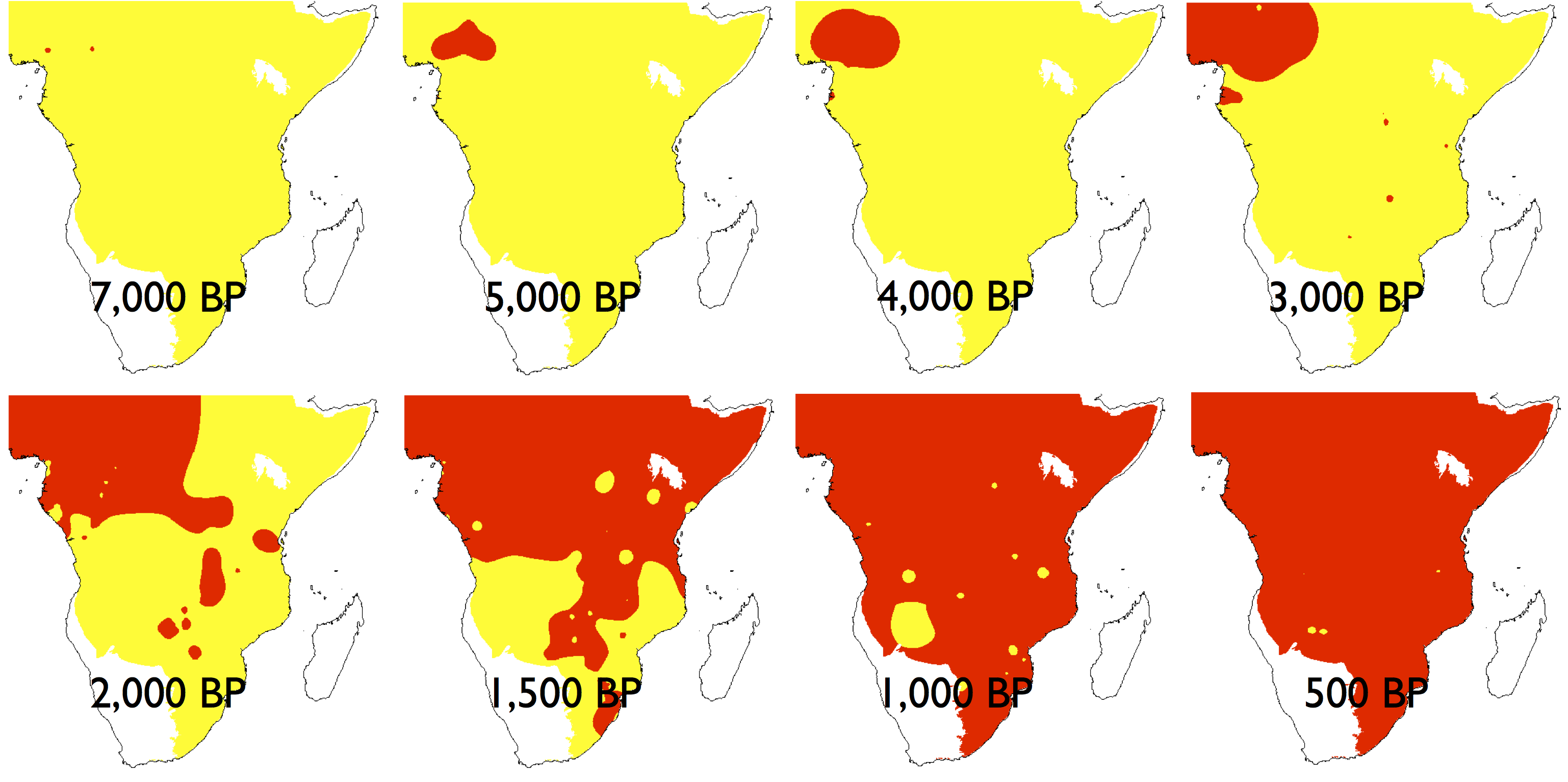

Supplement: Figure S2 — Isochron surface of the filtered subset. (TIFF) [file pone.0087854.s002.tiff]

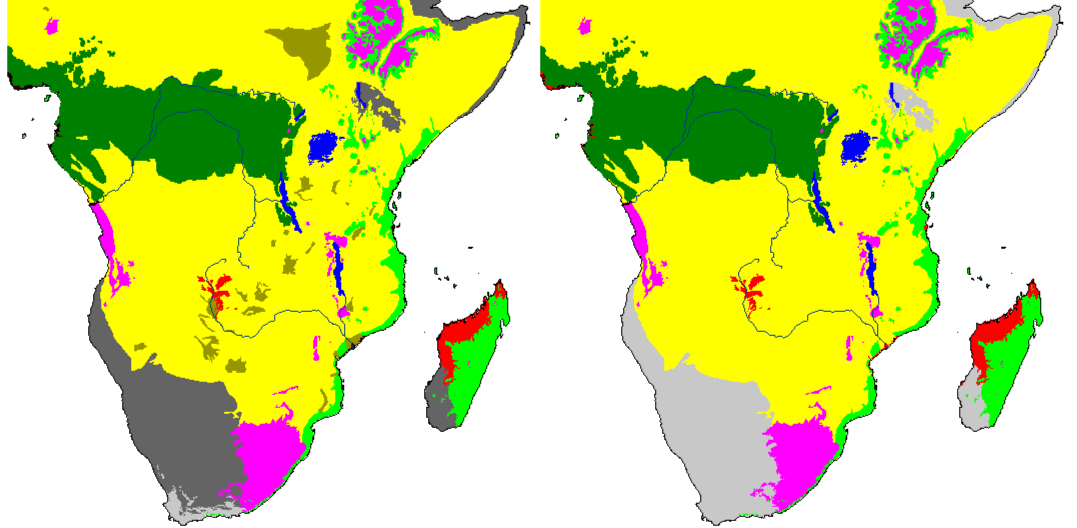

Supplement: Figure S3 — (left) All Olson biomes for the domain of interest; (right) biomes used for modelling, after aggregation. (TIFF) [file pone.0087854.s003.tiff]

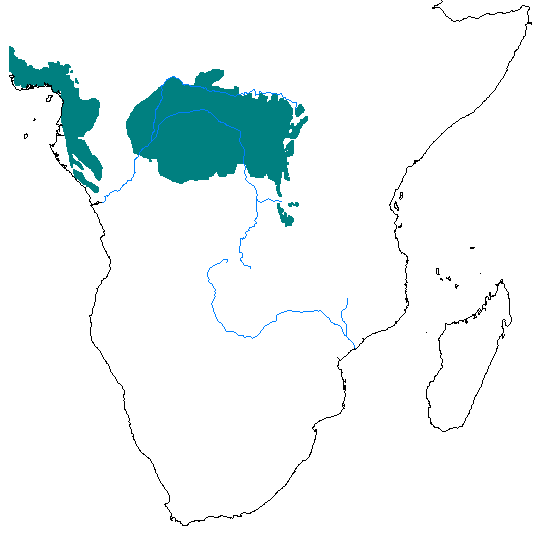

Supplement: Figure S4 — Map of the rainforest with savanna corridor, following Maley (2002). Also represented are the Congo and Zambezi rivers, and major tributaries, used in the modelling algorithms. (TIFF) [file pone.0087854.s004.tiff]
