# Supplementary material for: Modelling the Spread of Farming in the Bantu-Speaking Regions of Africa: An Archaeology-Based Phylogeography
Source: PLoS One. 2014 Jan 31;9(1):e87854. doi: 10.1371/journal.pone.0087854 (PMC3909244; doi:10.1371/journal.pone.0087854)
Supplement: Table S1 — Mechanisms of dispersal of farming and Bantu-languages in sub-Saharan Africa and how they are recognised, by Author. (DOCX) [file pone.0087854.s005.docx]

**Table S1**: Mechanisms of dispersal of farming and Bantu-languages in sub-Saharan Africa and how they are recognised, by Author.

| **Area** | **Author** | **Proxy used** | **Diffusion mechanism** | **Example** |
| --- | --- | --- | --- | --- |
| Central Africa  (Western Bantu expansion) | Vansina (1984) | Linguistics (genealogy of Bantu languages using vocabulary of modern day speakers of Bantu-languages) (using the work of Bastin *et al* 1983) + Archaeological evidence from West/Central Africa for Neolithic in this area. | Initial expansion of Bantu speaking farmers by demic diffusion. Followed later by cultural diffusion as population numbers grew and hunter-gatherers were outnumbered and adopted Bantu languages and farming or moved away. | “We may assume that the expansion was accompanied by migration of at least small numbers of people. Such an assumption has been proved for Eastern Bantu languages” (*ibid*: 133). And with regards to initial expansion of western Bantu speakers from area of modern day Cameroun, “…expansion began as a result of local population growth” (ibid: 133). “…the other widespread sites of the Gabon neolithic are probably to be attributed to the mass of Bantu speakers that was expanding south-eastwards from the Ogowe-estuary group. These migrants first found a rich savanna/ forest ecotone away from the upper Ogowe…” (ibid. 135). |
| Luangwa Valley, Eastern Zambia | Barham & Jarman (2005) | Archaeology (pottery, sorghum, radiocarbon dates | Demic diffusion | “A sequence of seven radiocarbon dates and associated pottery is highlighted as the first excavated evidence for the presence of early farmers in the Luangwa Valley” (*ibid*: 114). “The valley forms a biogeographical corridor that links eastern and southern Africa (Elton et al. 2003), and may have served as a natural route for the movement of human populations between these regions, including early farming communities” (*ibid*: 114). |
| Sub-equatorial Africa | Rexová *et al* (2006) | Linguistic | Demic diffusion | “Bantu expansion is an excellent example of prehistoric colonization on a continental scale. A fairly homogenous population of Bantu speaking people had spread from the northwest of the equatorial forest in Cameroon and Nigeria throughout Central, Eastern, and Southern Africa” ibid: 189 |
| Sub-equatorial Africa | Bostoen (2007) | Linguistic (more specifically studying 5,800 pottery-related terms from about 400 diﬀerent Bantu languages). | Demic diffusion and also the possibility of indigenous adoption and of bantu languages spreading with non-agriculturalists. | “From the second millennium BC onwards, the LSA industries give way to the typically SMA ceramic assemblages over the span of only a few centuries. All these elements favour the hypothesis of a technological revolution, which happened concurrently with the early spreading of Bantu languages in this area. Although technical diﬀusion and language shift amongst the pre-existing communities need to be taken into account, these processes were in all likelihood initiated by the migration of small Bantu speech communities” (*ibid*: 190).  “It is all the more important that the northern reflexes of *-ma`- (to make pottery) occur in languages descending from one or more of the primary offshoots of Proto-Bantu. Thus, the distribution of *-ma`- appears to confirm the historical link between these archaeological sites and the earliest West-Bantu language expansion.” (ibid: 188) |
| Sub-equatorial Africa | Phillipson (2005) | Archaeology | Demic diffusion | “The fact that so many important aspects of culture were introduced more-or-less together over such a wide area and so rapidly makes it highly probable that these innovations in sub-equatorial Africa were brought about as a result of the physical movement of substantial numbers of people” (ibid: 249). |
| Sub-equatorial Africa | Huffman (2007) | Archaeology | Demic diffusion | “Because of the vital relationship between language and material culture, ceramic style can be used to recognise and trace the movements of people even though their size, composition, linguistic scale and other characteristics are unknown” (ibid: 108). |
| Sub-equatorial Africa | Ribot (2004) | Craniometrics | Demic diffusion | “Morphological differences on an inter-regional level were identified in a more subtle manner than previously And they supported indirectly the morphological similarities (present within Bantu-speakers), which could result from a large-scale dispersal” (ibid: 20) |
| Sub-equatorial Africa | L’Abbé *et al.* (2006) | Craniometrics | Demic diffusion | “In fact, all the Bantu-speaking populations are the result of a relatively recent large-scale migration that produced a founder effect or homogenization process” (ibid: 24). |
| Sub-equatorial Africa | Mitchell (2002) | Archaeology | Demic and cultural diffusion | “The spread of farming southern Africa was clearly an uneven process, with several main movements apparent. To explain these we should look not only to demographic expansion, but also to the assimilation of hunter-gatherers into farming Communities” (ibid: 297) |
| Sub-equatorial Africa | Sinclair *et al* (1995) | Archaeology | Cultural diffusion | “A fundamental assumption is that we are dealing with the spread of iron-using Bantu-speaking agriculturalists, despite the fact that the contemporaneity of the different traits involved has at various times been challenged. A further unwarranted, and potentially dangerous assumption is the correlation of ceramic clusters with groups of people” (ibid: 12) |
| Western Branch (west-central Africa) | Clist (1989) | Pottery and other Archaeology | Demic diffusion | “If we associate these Neolithic expressions to human migrations, the new evidence enables us to postulate a slow migration of peoples, perhaps Bantu-speaking, through the evergreen forest and the coast. If further research in Lower Zaire shows the Ngovo industry to be the first such group south of the forest, then the rate of expansion must have been *ca* 1.2 kilometres a year. This agrees well with other models of similar small migrating groups”. (ibid: 80) |
| Sub-equatorial Africa | Scheinfeldt *et al*. (2010) | Genetics, MtDNA | Demic diffusion, sex-biased migration and gene flow due to patrilocality and/or polygyny. | “There is also a genetic signature of past population movements thought to be associated with the Bantu expansion” (ibid: 8934). “Conversely, the mtDNA haplogroup lineages in the same samples include lineages that are thought to have been present in the region before Bantu expansion” (ibid). |
| Sub-equatorial Africa | Pakendorf et al. (2011) | Molecular biology | Demic diffusion | “Contrary to some assumptions by historians and cultural anthropologists, the genetic data speak in favor of an actual movement of peoples during the expansion of the Bantu languages over Africa, rather than a spread through language and culture shift.” (ibid: 50). |
| Central Zambia | Robertson (2000) | Archaeology | Cultural diffusion | “. The Mulungushi research suggests the adoption of EIA traits in Zambia was a process involving the autochthonous people rather than an event, such as a migration of new people.” (ibid: 179) |
| Sub-equatorial Africa | De Filippo et al. (2012) | Genetic and linguistics | Demic diffusion | “Our analyses primarily indicate that the dispersal of Bantu languages was coupled with the movement of people (i.e. demic diffusion), as demonstrated by the lower genetic distances among Bantu populations when compared with those between Bantu and all the other major ethno-linguistic groups, as well as by the reduction of mtDNA and Y-chromosomal diversity proportional to the distance from Bantu homeland”. (ibid: 3262) |

**References**

Barham, L. & Jarman, C.L., 2005. New Radiocarbon Dates for the Early Iron Age in the Luangwa Valley, Eastern Zambia. *Azania: Archaeological Research in Africa* 40 (1): 114-121.

Bastin Y, Coupez A, Mann M., 1999. Continuity and divergence in the Bantu languages: perspectives from a lexicostatic study. Tervuren, Belgium: Museée royal de l’Afrique centrale.

Bostoen, K., 2007. Pots, words and the Bantu problem: on lexical reconstruction and early African history. *Journal of African History* 48: 173-99.

Clist, B., 1989. Archaeology in Gabon, 1886-1988. African Archaeological Review 7, 59-95.

De Filippo, C., Bostoen, K., Stoneking, M. & Pakendorf, B., 2012. Bringing together linguistic and genetic evidence to test the Bantu expansion. *Proc. R. Soc. B* 279: 3256-3263.

Huffman, T.N., 2007. *Handbook to the Iron Age: the Archaeology of pre-colonial farming societies in southern Africa*. Scottsville: University of KwaZulu-Natal Press.

L’Abbé, E.N., Ribot, I & Steyn, M., 2006. A craniometric study of the 20th century Venda. *South African Archaeological Bulletin* 61(183): 19-25.

Maley, J., 2002. A Catastrophic Destruction of African Forests about 2,500 Years Ago Still Exerts a Major Influence on Present Vegetation Formations. *IDS Bulletin* 33(1): 13-30.

Mitchell, P., 2002. *The Archaeology of Southern Africa*. Cambridge: Cambridge University Press.

Olson, D.M., E. Dinerstein, E.D. Wikramanayake, et al., 2001. Terrestrial Ecoregions of the World: A New Map of Life on Earth (PDF, 1.1M) *BioScience* 51: 933-938.

Pakendorf, B., Bostoen, K. & de Filippoa, C., 2011. Molecular Perspectives on the Bantu Expansion: A Synthesis. *Language Dynamics and Change* 1:50–88.

Phillipson, D.W., 2005. African Archaeology. Cambridge: Cambridge University Press.

Rexová K, Bastin Y, Frynta D., 2006. Cladistic analysis of Bantu languages: a new tree based on combined lexical and grammatical data. *Naturwissenschaften* 93: 189-194.

Ribot, I., 2004. Differentiation of modern sub-Saharan African populations: craniometric interpretations in relation to geography and history. *Bull Mem Soc Anthropol Paris* 16:143-165.

Robertson, John H., 2000. Early Iron Age Archaeology in Central Zambia. *Azania: Archaeological Research in Africa*, 35 (1):147-182.

Scheinfeldt, L.B., Soi, S. & Tishkoff, S.A., 2010. Working towards a synthesis of archaeological, linguistic, and genetic data for inferring African population history. *PNAS* 107 (suppl. 2): 8931-8938.

Sinclair, P.J.J., Shaw, T. & Andah, B., 1995. Introduction. In: *The Archaeology of Africa: food, metals and towns*. Shaw, T., Sinclair, P.J.J., Andah, B. & Okpoko, A. (eds) 1-31. London: Routledge.

Vansina, J., 1984. Western Bantu expansion. *The Journal of African History*. 25(2): 129-145.
