# Supplementary material for: Modelling the Spread of Farming in the Bantu-Speaking Regions of Africa: An Archaeology-Based Phylogeography
Source: PLoS One. 2014 Jan 31;9(1):e87854. doi: 10.1371/journal.pone.0087854 (PMC3909244; doi:10.1371/journal.pone.0087854)
Supplement: Table S2 — Database used in the analysis, after the site selection process. (DOCX) [file pone.0087854.s006.docx]

**Table S2**: Database used in the analysis, after the site selection process.

| **Site** | **Latitude** | **Longitude** | **Uncal BP** | **St** **Dev** | **Mean Cal BP** | **Stream** |
| --- | --- | --- | --- | --- | --- | --- |
| Feti la Choya | -13.43 | 15.88 | 1240 | 100 | 1153.87 | Western |
| Furi I Mine | -7.37 | 20.67 | 1800 | 80 | 1729.69 | Western |
| Kitala II | -9.03 | 13.08 | 1230 | 60 | 1159.15 | Western |
| Museque Cave | -10.83 | 19.17 | 465 | 80 | 485.85 | Western |
| Bisoli | -21.03 | 27.57 | 1304 | 48 | 1223.66 |  |
| Broadhurst | -24.53 | 25.95 | 590 | 60 | 593.49 | Western |
| Taukome (lower levels) | -29.86 | 32.72 | 1253 | 57 | 1178.85 |  |
| Toteng I | -20.42 | 22.95 | 1820 | 50 | 1754.19 |  |
| Mubuga V | -3.40 | 30.02 | 3160 | 145 | 3370.79 | Eastern |
| Campo | 2.33 | 9.82 | 960 | 50 | 860.79 | Western |
| Mbengue | 3.78 | 10.15 | 1010 | 25 | 927.88 | Western |
| Obobogo | 3.82 | 11.50 | 3625 | 165 | 3985.59 | Western |
| D_guesse | 12.05 | 14.87 | 3350 | 270 | 3639.33 |  |
| Doulo Igzawa 1 | 11.12 | 14.17 | 2500 | 60 | 2571.32 |  |
| Nassarao | 9.35 | 13.45 | 5330 | 350 | 6125.83 |  |
| Shum-Laka | 5.85 | 10.07 | 6980 | 260 | 7842.48 |  |
| Tazunu Zupaya | 5.97 | 15.58 | 6700 | 140 | 7572.84 |  |
| Djambala | -2.55 | 14.75 | 2300 | 100 | 2337.79 |  |
| Mpassa farm | -4.30 | 14.27 | 660 | 80 | 620.57 | Western |
| Tchissanga Ouest | -4.53 | 11.80 | 2880 | 90 | 3030.65 |  |
| Ditouba | -3.27 | 10.50 | 1200 | 60 | 1126.76 |  |
| Lac Bleu de Mouila | -1.50 | 11.17 | 1997 | 42 | 1950.84 | Western |
| Makokou | 0.57 | 12.85 | 2150 | 70 | 2154.59 |  |
| Mbilap_ 4 | -0.88 | 9.15 | 2455 | 47 | 2539.80 |  |
| Moanda II | -1.56 | 13.18 | 2148 | 37 | 2160.32 | Western |
| Mont Brazza | -0.13 | 11.59 | 3560 | 75 | 3853.33 |  |
| Oyem 2 | 1.57 | 11.57 | 2259 | 44 | 2249.82 |  |
| Rivire Denis | 0.32 | 9.35 | 4810 | 80 | 5527.07 |  |
| Enkapune Ya Muto (GtJil2) | -0.83 | 36.15 | 1295 | 140 | 1205.13 | Eastern |
| Ganga GpJa 17 | 0.33 | 34.25 | 1760 | 160 | 1688.13 | Eastern |
| Kithiru GpJp 35 | 0.42 | 37.77 | 1625 | 155 | 1557.72 | Eastern |
| Kwale Forest | -4.18 | 39.43 | 1850 | 115 | 1779.18 | Eastern |
| Kwamboo | -1.00 | 37.25 | 1746 | 88 | 1669.48 | Eastern |
| Lanet II, Nakuru | -10.30 | 36.12 | 375 | 100 | 397.82 | Eastern |
| Manda Island | -2.22 | 40.97 | 1430 | 110 | 1347.04 | Eastern |
| Chaminade | -9.95 | 33.88 | 2100 | 80 | 2098.50 | Eastern |
| Mtuzi Hill Rockshelter (DZ126) | -14.27 | 34.18 | 1646 | 41 | 1541.82 | Eastern |
| Phopo Hill | -11.12 | 33.65 | 1676 | 83 | 1583.67 | Eastern |
| Chibuene | -22.03 | 35.32 | 1229 | 35 | 1159.84 | Eastern |
| Hola hola | -21.30 | 34.31 | 1060 | 50 | 981.38 | Eastern |
| Massingir | -23.15 | 32.00 | 1030 | 40 | 946.97 | Eastern |
| Matola IV | -25.95 | 32.45 | 1856 | 46 | 1786.62 | Eastern |
| Dikundu | -18.10 | 21.67 | 120 | 50 | 140.10 | Western |
| Kapako | -20.50 | 17.25 | 1110 | 50 | 1027.90 | Western |
| Vungu Vungu | -17.88 | 19.85 | 290 | 45 | 358.29 | Western |
| Kabacusi | -2.08 | 29.67 | 2815 | 165 | 2977.57 | Eastern |
| Mucucu II/3 lioness shelter | -1.53 | 30.50 | 2380 | 270 | 2425.52 | Eastern |
| Broederstroom, 24/73 K | -25.75 | 27.83 | 1520 | 110 | 1438.91 |  |
| Diamant | -23.75 | 28.25 | 1380 | 50 | 1296.75 |  |
| Enkwazini | -28.18 | 32.51 | 1605 | 38 | 1483.45 |  |
| Klein Afrika | -22.83 | 29.92 | 1590 | 33 | 1473.05 |  |
| Mabveni | -20.37 | 30.47 | 1770 | 120 | 1699.18 |  |
| Mpame | -32.10 | 29.05 | 1264 | 33 | 1207.07 |  |
| Ntsitsana, Pit 1 | -31.07 | 29.20 | 1290 | 50 | 1212.77 |  |
| Shongweni Waterworks Park | -29.87 | 30.72 | 2030 | 45 | 1992.81 |  |
| Silver Leaves | -23.92 | 30.14 | 1692 | 24 | 1599.01 |  |
| Langubhela | -26.28 | 31.17 | 1620 | 50 | 1506.98 |  |
| Amboni Cave | -5.07 | 39.05 | 1590 | 120 | 1512.40 |  |
| Bombo Kaburi | -4.27 | 38.00 | 1730 | 115 | 1638.99 |  |
| Ivuna | -8.43 | 32.48 | 646 | 56 | 610.43 |  |
| Katuruka | -1.45 | 31.77 | 2450 | 81 | 2533.69 |  |
| Kilwa Kisiwani | -8.95 | 39.52 | 1825 | 110 | 1749.91 |  |
| Kwelikwiji | -6.10 | 37.55 | 3130 | 60 | 3346.35 |  |
| Limbo | -7.47 | 39.08 | 2003 | 50 | 1959.29 | Eastern |
| Mumba | -3.50 | 35.33 | 1780 | 80 | 1702.51 |  |
| Mwika | -3.27 | 37.58 | 1700 | 330 | 1663.13 |  |
| Pwaga | -5.07 | 30.38 | 1530 | 160 | 1454.17 |  |
| Bigo | 0.38 | 29.90 | 496 | 54 | 537.31 |  |
| Chobi | 2.25 | 32.20 | 1660 | 130 | 1574.65 |  |
| Bokele | -0.08 | 18.58 | 2290 | 70 | 2288.99 |  |
| Boma | -5.85 | 13.07 | 1870 | 105 | 1801.38 |  |
| Boso-Njafo | 1.00 | 19.17 | 2285 | 50 | 2267.11 |  |
| Imbonga | -0.80 | 19.77 | 2160 | 90 | 2151.57 |  |
| Malemba-Nkulu | -8.22 | 26.78 | 1318 | 52 | 1237.11 |  |
| Maluba | 2.83 | 18.55 | 1931 | 47 | 1878.13 |  |
| Munda | 1.08 | 17.25 | 1965 | 25 | 1914.83 |  |
| Naviundu River | -11.65 | 27.53 | 1571 | 37 | 1460.65 |  |
| Nsele | -4.25 | 15.53 | 2190 | 90 | 2182.28 |  |
| Ntadi ntadi Cave | -5.77 | 14.54 | 2155 | 60 | 2162.68 |  |
| Pikunda | -0.50 | 16.67 | 1980 | 100 | 1945.67 |  |
| Chowo River Site | -24.21 | 31.20 | 1635 | 28 | 1520.53 | Eastern |
| Isami Pati mound/ Isamu Pati Mound | -17.07 | 26.33 | 1852 | 100 | 1783.22 |  |
| Kalambo Falls | -8.58 | 31.25 | 2730 | 40 | 2827.44 |  |
| Kamnama | -13.53 | 32.85 | 1600 | 110 | 1520.38 |  |
| Kamusongolwa Kopje | -13.45 | 25.85 | 800 | 100 | 754.91 |  |
| Kangonga | -13.07 | 28.67 | 1610 | 115 | 1525.74 |  |
| Kansanshi Hill | -11.67 | 26.50 | 1428 | 62 | 1340.66 |  |
| Kapwirimbwe | -15.38 | 28.37 | 1505 | 55 | 1403.39 |  |
| Kumadzulo | -17.62 | 25.53 | 2970 | 105 | 3135.96 |  |
| Lubusi | -14.37 | 23.82 | 1070 | 60 | 993.25 |  |
| Makwe Shelter | -14.40 | 31.93 | 1730 | 110 | 1653.32 |  |
| M'teteshi 1 | -14.28 | 28.60 | 1986 | 38 | 1938.30 |  |
| Mufulwe | -13.97 | 29.58 | 2170 | 50 | 2188.50 |  |
| Mumbwa | -14.98 | 27.08 | 2250 | 160 | 2279.08 |  |
| Mwanamaimpa | -15.98 | 26.12 | 1258 | 86 | 1160.72 |  |
| Nakapapula | -12.82 | 30.62 | 3280 | 90 | 3517.50 |  |
| Ndonde | -16.73 | 27.40 | 2370 | 115 | 2447.97 |  |
| Salumano | -17.20 | 24.12 | 2330 | 65 | 2372.62 |  |
| Situmpa | -16.25 | 25.12 | 2171 | 38 | 2199.96 |  |
| Bambata Cave Series | -20.50 | 28.41 | 2850 | 60 | 2976.04 |  |
| Coronation Park I | -17.83 | 31.10 | 1240 | 100 | 1148.05 |  |
| Kinsale Farm | -18.60 | 26.33 | 1410 | 95 | 1324.34 |  |
| Malapati | -22.05 | 31.42 | 1110 | 100 | 1043.07 |  |
| Mutema's Sacred Grove/Muntema's grove, Melsetter | -19.98 | 32.55 | 771 | 72 | 721.28 |  |
| Naba (Ndaba) | -16.87 | 29.68 | 1310 | 50 | 1231.42 |  |
| Zimbabwe, Fort Victoria | -21.05 | 31.52 | 1269 | 54 | 1192.89 |  |
| Ziwa Farm | -18.20 | 32.67 | 1650 | 100 | 1559.24 |  |
