# Supplementary material for: Modelling the Spread of Farming in the Bantu-Speaking Regions of Africa: An Archaeology-Based Phylogeography
Source: PLoS One. 2014 Jan 31;9(1):e87854. doi: 10.1371/journal.pone.0087854 (PMC3909244; doi:10.1371/journal.pone.0087854)
Supplement: Table S3 — Number of sites and dates on the full database, per country and area. (DOCX) [file pone.0087854.s007.docx]

**Table S3**: Number of sites and dates on the full database, per country and area.

| **Country (land area, km^2^)** | **No. of Sites** | **No. of Dates** | **Sites/ 100,000 km^2^** |
| --- | --- | --- | --- |
| Angola (1,246,700) | 10 | 11 | 0.80 |
| Botswana (581,041) | 9 | 13 | 1.55 |
| Burundi (27,830) | 3 | 9 | 10.78 |
| Cameroon (475,442) | 34 | 106 | 7.15 |
| Central African Republic (622,984) | 7 | 19 | 1.12 |
| Democratic Republic of the Congo (2,345,410) | 22 | 67 | 0.94 |
| Gabon (267,668) | 35 | 74 | 13.08 |
| Kenya (580,367) | 19 | 34 | 3.27 |
| Malawi (118,484) | 12 | 19 | 10.13 |
| Mozambique (801,590) | 6 | 20 | 0.75 |
| Namibia (825,418) | 3 | 3 | 0.36 |
| Republic of Congo (342,000) | 6 | 13 | 1.75 |
| Rwanda (26,798) | 9 | 10 | 33.58 |
| South Africa (1,221,037) | 48 | 116 | 3.93 |
| Swaziland (17,364) | 2 | 5 | 11.52 |
| Tanzania (945,087) | 16 | 47 | 1.69 |
| Uganda (236,040) | 3 | 5 | 1.27 |
| Zambia (752,614) | 48 | 167 | 6.38 |
| Zimbabwe (390,757) | 39 | 66 | 9.98 |
| **TOTAL** | **331** | **804** | **Average=6.32** |
