# Supplementary material for: Modelling the Spread of Farming in the Bantu-Speaking Regions of Africa: An Archaeology-Based Phylogeography
Source: PLoS One. 2014 Jan 31;9(1):e87854. doi: 10.1371/journal.pone.0087854 (PMC3909244; doi:10.1371/journal.pone.0087854)
Supplement: Text S1 — Supporting information on methods used. (DOCX) [file pone.0087854.s008.docx]

**Modelling the spread of farming in the Bantu-speaking regions of Africa: an archaeology-based phylogeography**

Thembi Russell, Fabio Silva and James Steele

**Supporting Information - Methods**

1. **Site Selection**

As an attempt to improve the accuracy of the fitting process it is common to filter out sites that do not correspond to first arrivals. This is a process that can be done qualitatively, by appraising each individual date and site, or quantitatively by binning with respect to some variable. A typical example of the latter involves picking out only the oldest site on each equal-size bin of the distance to the assumed dispersal source. We will call this method ‘radial 1D binning’ as the binning is done on a single dimension: the distance. This methodology, however, is particularly impaired when working on anisotropic distributions over vast land surfaces as a younger site located in a different direction to another, but with the same distance from the putative source, will be excluded, thus filtering out potentially valid data.

One way around this issue is to consider a grid of equally sized square cells, the bins, and then keep only the oldest site on each cell. Despite its advantages this approach would introduce ambiguities related to the laying out of an arbitrary grid, namely questions on how using different grid reference points (which would affect the location of the grid cells) affects the results of the binning. The question of how to deal with data located on the border between two cells would also affect the binning output.

To work around these issues and lose none of its advantages a 'radial 2D binning' technique was developed. The “gridding” is done radially from each datapoint, i.e. a circle of a specified radius (the bin size) at each site of the database is considered. The age of all datapoints within each “grid circle” is checked and the oldest site tagged. This is done for all datapoints, after which the ones that haven’t been tagged at all are filtered out. The whole process is repeated several times to ensure a thorough filtering. This effectively results in a selection of the oldest sites that are at least one bin size apart. This methodology has the advantage that, unlike 'radial 1D binning', it is no longer attached to a putative dispersal origin (at distance zero), thus being independent of any such assumptions, besides not being blind to anisotropic distributions of radiometric dates.

The bin size (i.e. the radius of the “grid circle”) was estimated to be the resolution of the dispersal wavefront, which is tied to the radiocarbon uncertainty. Because there is an uncertainty in the radiocarbon dates of all sites, which averages to about 100 years, two sites a hundred kilometres distant wouldn’t be able to be distinguished by a typical wavefront of 1 km/yr speed. Using these values we have implemented 100km radius bins. The same reasoning applies for two sites whose mean radiocarbon dates fall within the 100 year standard deviation and whose distance is smaller that the bin size of 100 km. All such sites were also tagged in our technique, and thus kept.

1. **Cost Distance Modelling**

To simulate the diffusive processes in a heterogeneous surface a modified Fast Marching Method (FMM) was used. This method computes the cost distance of an expanding front at each point of a discrete lattice or grid from the source of the diffusion. The FMM was originally devised by Sethian (1999) to simulate uniformly expanding fronts whose motion is described by the Eikonal equation. The algorithm works outwards from an initial condition, the source of the expanding front, by tracking a narrow band around the front. The Eikonal equation, which gives the arrival time for a given grid point, is solved by a second-order finite-difference approximation, which was found to be quite accurate. This algorithm was further modified by Kobayashi and Sugihara (2001) who applied it to crystal growth scenarios where each region internal to a diffusive front (a patch, or crystal in their context) acts as an obstacle to all other fronts.

Another modification was developed by two of the present authors (Silva and Steele 2012) to further generalize the algorithm to accommodate scenarios in which the different diffusive processes can begin at different times. In addition, and for current purposes, this algorithm was further modified to react to a heterogeneous domain containing surface patches (ecoregions, rivers, etc.) that will affect the rate of spread in said patches. This was achieved by attributing a friction value to these patches which is then used in the calculation of speed locally (see equation (2) in Silva and Steele, 2012). In this way corridors, where the rate of spread is boosted, or obstacles, where the spread is slowed down, are included in the models and the cost distance estimation. This methodology is discussed in depth by Silva and Steele (2012, submitted).

1. **Shortest Path Trees**

The obtained cost distance surface can then be used to derive shortest paths (“least-cost paths”) from the origin to any geographical location, as a function of the friction weights assigned to each class of geographical feature. Points where least-cost paths meet can be considered as nodes on a tree, with branches corresponding to the archaeological instances, and the entire least-cost path network represented as a phylogenetic tree that epitomises the dispersal history of a particular model – a “shortest path” or “dispersal tree”. The information required to reconstruct the least-cost path network as a dispersal tree, namely the branches, nodes and distances between them, was extracted and converted into the required format, again using a purpose-built MATLAB algorithm. Its results were compared to the outputs of similar GIS algorithms (namely the *r.drain* module of GRASS GIS) with good congruence. This methodology allows one to test whether purely radiocarbon-based dispersal models can also account for cultural features, such as languages or pottery styles. This can be done by, for each model, reconstructing the dispersal tree for a given set of archaeological sites for which pottery styles (Western stream or Eastern stream) have been identified. This methodology is introduced in Silva and Steele (submitted).

1. **Genetic Algorithms**

In order to let the archaeological data speak for itself and not impose any prior constraints on the models, we also attempted to obtain the parameter set that provides the best fit to the radiocarbon dataset independently of prior hypotheses in the literature. The problem is one of optimization, i.e. of finding the set of parameters that maximizes a fitness function: in this case the correlation coefficient. Fully exploring the parameter space is a computationally slow process so, in order to quickly and effectively find the best-fit model we decided to implement a Genetic Algorithm (GA henceforth). GAs are optimization and search techniques based on the Darwinian principle of natural selection, as well as genetics. It mimics the natural processes of reproduction, including selection, mating with crossover and mutation, in order to ‘evolve’ a best-fit parameter set out of a random population of parameters. GAs were developed originally by Holland (1975) and, particularly since the 1980s, they rose in popularity due to their usefulness for function optimization and other applications. They have since become standard techniques in several disciplines, including bioinformatics, computational science, mathematics and engineering (Haupt and Haupt 2004).

A typical GA run starts with a random population of models (i.e. a set of models with random values for the parameters) whose fitness is evaluated by some function (in our case the regression analysis). The best-fit models are then copied to the next generation unscathed (cloned), whereas less fit models are discarded. To keep the population size constant, the best-fit models are also allowed to reproduce. This involves the genetic principle of crossover, in which both parent models give only a part of their parameter set to the child model. Mutation can then occur on any model of the new generation, except for the very best one. This process is iterated several times until a certain condition is met. Crossover and mutation are controlled by fixed rates and are essential to ensure that the GA doesn’t get stuck on a local maximum of the fitness function but instead sample enough of the parameter space to lock-on to a global maximum. After several generations the population begins to converge on the parameter set that maximizes the fitness which in this case, is given by the correlation coefficient. This GA application to archaeology was developed specifically for this project using MATLAB. The GA parameters used to obtain the presented results were the following: population size was kept constant at 10; each generation kept 50% of the previous one, the other half was populated by heuristic crossover (Haupt and Haupt 2004, 58) and a mutation rate of 20% was used. The GA was allowed to run for 300 generations, and convergence was then confirmed by checking the variation in the best-fit models of the last hundred generations, and whether the parameter space had been sufficiently sampled.

**References**

Haupt, R. L. and Haupt, S. E., 2004. *Practical Genetic Algorithms*, 2nd edn. New Jersey: John Wiley & Sons.

Holland, J. H., 1975. *Adaptation in Natural and Artificial Systems*. Ann Arbor: University of Michigan Press.

Kobayashi, K. and Sugihara, K., 2001. Crystal voronoi diagram and its applications (algorithm engineering as a new paradigm),
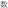

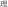

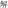

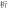

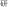

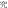

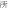

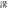

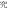

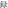
1185:109–119, <http://hdl.handle.net/2433/64634>.

Sethian, J. A., 1999. *Level set methods and fast marching methods: Evolving interfaces in computational geometry, fluid mechanics, computer vision, and materials science,* 2nd edn. Cambridge: Cambridge University Press.

Silva, F. and Steele, J., 2012. Modeling boundaries between converging fronts in prehistory. *Advances in Complex Systems* 15 (1-2), 1150005.

Silva, F. and Steele, J., submitted. New Methods for Reconstructing Dispersal Rates and Routes from Large-scale Radiocarbon Databases. *Journal of Archaeological Science.*
